# Glycan signal enhancement by ammonium fluoride doping and electrospray diverting

**DOI:** 10.1039/d6an00090h

**Published:** 2026-03-23

**Authors:** Seth M. Eisenberg, David C. Muddiman

**Affiliations:** a Biological Imaging Laboratory for Disease and Exposure Research (BILDER), Department of Chemistry, North Carolina State University Raleigh NC 27695 USA dcmuddim@ncsu.edu

## Abstract

By combining ammonium fluoride electrospray doping with an electrospray diverter, signals of *N*-linked glycans were significantly enhanced. The addition of NH_4_F into the electrospray increases signal by deprotonating neutrals and creating [M − H]^−^ ions. The electrospray diverter can prevent excess background ions from entering the mass spectrometer ion trap, reducing ion suppression. Utilizing these techniques simultaneously resulted in a synergistic effect, increasing signal more than demonstrated previously by either technique. Cleaved *N*-linked glycans analyzed from a solution showed an average 9-fold signal increase while cleaved *N*-linked glycans from tissue experienced an average 27-fold increase in signal. These increases showed no statistical differences when considering their *m*/*z*, adduct, or specific monosaccharides, suggesting that this technique is highly robust and agnostic to analyte characteristics.

## Abstract 2

By combining ammonium fluoride electrospray doping with an electrospray diverter, signals of *N*-linked glycans were significantly enhanced.

## Graphical abstract

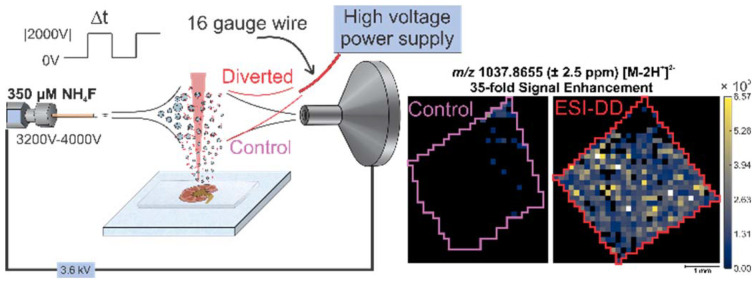

## Introduction

Mass spectrometry imaging (MSI) enables spatial mapping of a sample of interest, observing hundreds to thousands of ions simultaneously. Over the past 20 years, the field of mass spectrometry has seen the rise of ambient ionization, where ions are generated without a vacuum.^1^ A variety of sources utilize this technique, divided into 3 main categories: liquid extraction (*e.g.*, desorption electrospray ionization (DESI),^2^ nano-DESI,^3^ secondary electrospray ionization (SESI)^4,5^), plasma desorption (*e.g.*, atmospheric pressure chemical ionization (APCI),^6^ direct analysis in real time (DART)^7^), and laser ablation (*e.g.*, matrix-assisted laser desorption/ionization (MALDI),^8^ infrared matrix-assisted laser desorption electrospray ionization (IR-MALDESI)^9^). Ambient ionization provides benefits including reduced sample preparation, higher throughput, and it creates a more representative environment to analyze tissues.^1^ However, sources operating in the atmosphere can collect ions unrelated to the sample of interest, known as background ions.^10^ These harmful signals can result in ionization suppression and confounding peaks in the mass spectra.

Glycosylation is a common post-translational modification (PTM) that results in the addition of glycans, or sugars, to the protein backbone, wherein over 50% of mammalian proteins are glycosylated. These glycans play vital biological functions including cell signaling,^11^ adhesion,^11^ trafficking,^12^ and protein stability.^13^ Importantly, abnormal or disrupted glycosylation patterns are implicated in a variety of autoimmune diseases, cancers, and infectious diseases.^13^*N*-linked glycans share a common pentasaccharide core and form only on specific amino acid motifs.^14^ They can be specifically cleaved from proteins using enzymes, enabling their detection by mass spectrometry. However, *N*-glycans have low ionization efficiency and their signals are significantly reduced by background ions. The ability to detect and study glycans and their localization in a tissue is highly important due to their pervasiveness and biological importance.

Recently, an electrospray diverter was developed and optimized to enable the significant reduction of background ions.^15^ The operating principle behind this technique is based on controlling the ions allowed into the ion trap of a mass analyzer. For many trapping-based mass analyzers, including Orbitraps, ions require a time-of-flight to move through the mass spectrometer to reach the ion routing multipole (IRM) and be trapped before they are measured. During this time, additional ions, unrelated to the sample of interest, can enter and be trapped as well, causing signal suppression. Using the diverter, ions generated after the sample-related ions have entered the mass spectrometer can be diverted away from the inlet. This was shown to reduce background signals by up to 4-fold, leading to increased sample-related signal up to an order of magnitude from a solution of cleaved *N*-linked glycans.^15^ Previously, the C-trap opening and timing was optimized to prevent collecting background signal before the tissue related ions entered the MS.^16^ In contrast, the ESI diverter works after this step, preventing excess ions from entering the system after tissue related ones have already been collected and while they are guided through the MS to the trapping cell. This technique was more impactful for larger *m*/*z* such as glycans or proteins, as they require a significantly longer time-of-flight to reach the trapping cell.

One common method to improve the detection of low abundant analytes is by improving signal-to-noise ratio and raising signals above the limit of detection (LOD). The addition of metals or salts to an electrospray, known as electrospray doping, can either increase signal abundance^17–22^ or provide unique structural information.^23^ Upon adding NH_4_F, the electronegative and basic fluoride ion is hypothesized to capture protons from neutral analytes to generate increased [M − H]^−^. This was first demonstrated in MALDI-MSI^21^ and nano-DESI,^22^ and recently optimized for IR-MALDESI,^24^ as well as showcasing its use for quantitative MSI.^25^ After concentration optimization for glycan detection, up to a 4-fold increase in signal was observed, as well as a 30% increase in detection frequency.

Due to the orthogonality of these techniques for signal enhancement, herein they are easily combined, without any interfering mechanisms. Using IR-MALDESI, NH_4_F doped ESI and ESI diverting, referred to as ESI-DD, are used simultaneously to demonstrate a synergistic effect, resulting in significant signal increases for glycans across a wide *m*/*z* range.

## Experimental

### Materials

LC-MS grade water, acetonitrile (ACN), and acetic acid were purchased from Thermo Fisher Scientific (Nazareth, PA, USA). Ammonium fluoride (>98.0%) was purchased from Sigma Aldrich (St Louis, MO, USA). Nitrogen gas was purchased from Arc3 gases (Raleigh, NC, USA).

### Glycan preparation

*N*-Linked glycans were prepared from bovine fetuin (Sigma Aldrich) as described previously.^26^ In short, 250 µg of bovine fetuin was loaded onto a 10 kDa molecular weight cutoff filter with dithiothreitol (Sigma Aldrich) and 100 mM ammonium bicarbonate (Sigma Aldrich) to denature. The proteins were then alkylated with iodoacetamide (Sigma Aldrich) followed by cleavage of *N*-linked glycans using 1000 units of PNGase F PRIME-LY (Bulldog Bio, NJ, USA). The proteins were incubated overnight at 37 °C before elution and drying in a vacuum desiccator. The cleaved *N*-linked glycans were resuspended in LC-MS grade water and stored at −20 °C until analysis.

Formalin-fixed paraffin-embedded (FFPE) human kidney samples were obtained from the Drake laboratory at the Medical University of South Carolina. The samples were sectioned to 3 µm by a rotary microtome (HM355S, Epredia, Kalamazoo, MI, USA) before mounting onto uncharged slides. These slides were prepared for MSI following published protocol.^27^ In brief, the wax was melted for 1 h at 60 °C before a series of dewaxing washes consisting of 2× xylenes (3 min), 2× 100% ethanol (1 min), 1× 95% ethanol (1 min), 1× 70% ethanol (1 min), and 2× 100% LC-MS grade water (3 min). The dewaxed slides were vacuum dried and underwent antigen retrieval using citraconic acid buffer and heat. Afterwards, the buffer was exchanged for LC-MS water and the slide dried again. *N*-Linked glycans were cleaved by 100 µg mL^−1^ of PNGase F PRIME-LY sprayed using a TM-Sprayer (HTX Imaging, Carrboro, NC, USA) and a 2 hour incubation in a humidity chamber (95% relative humidity). The slides were stored at −80 °C until imaged and analyzed by IR-MALDESI.

### IR-MALDESI-MSI analysis

The next-generation IR-MALDESI source^28^ was utilized for all droplet and tissue imaging experiments. As previously described, it was interfaced with an Orbitrap Exploris 240 mass spectrometer (Thermo Fisher Scientific, Bremen, Germany).^9^ For tissue imaging, the slide-mounted sample was placed on a Peltier-cooled stage inside a humidity-controlled enclosure. The enclosure was purged with inert nitrogen gas to reach <12% relative humidity (RH) before cooling the stage to −10 °C. After tissue cooling and temperature stabilization, the nitrogen flow was stopped and the enclosure was opened to allow an increase in RH, thereby depositing a thin ice layer on the tissue over the course of ∼4 minutes. After ice deposition, the enclosure was shut to prevent further ice growth. For solution-based experiments to analyze glycans cleaved from bovine fetuin, a well plate was positioned on the stage, and no external matrix was applied. In either case, a burst mode mid-IR laser (JGM Associates, Burlington, MA, USA) was fired at 2970 nm at varying pulses per burst to ensure at least 1.5 mJ reached the sample surface to resonantly excite the O–H stretching vibrational mode of the endogenous water within the tissue and the exogenously applied ice matrix, if present. This causes ablation of neutral molecules, which partition into the orthogonal electrospray plume before being ionized in an electrospray-like mechanism.^29^ The control ESI solvent consisted of 50% ACN, 1 mM formic acid in water, flowing at 1.2 µL min^−1^ with an applied voltage of −3.5 kV.^30^ When the ESI was doped, it was composed identically, except for the addition of 350 µM NH_4_F.

Data were collected in negative, profile mode at a resolving power of 240 000 FWHM at *m*/*z* 200 with the EASY-IC internal calibrant fluoranthene ([M˙^+^] *m*/*z* 202.0777) enabled. The automatic gain control function was disabled, holding the injection time constant at 90 ms. Mass spectra were recorded from *m*/*z* 500 to *m*/*z* 2000 with the RF ratio equal to 4 and the S-lens RF value set at 70%.

### Electrospray diverter

The electrospray diverter was set up and positioned as described previously.^15^ Briefly, an Optorelay was connected to a high voltage power source and a small wire placed directly above the inlet to the mass spectrometer. Controlled by an Arduino,^31^ the voltage was deactivated at precise timing events to ensure sample related ions enter the mass spectrometer and excess background ions are not allowed to enter during the remainder of the ion injection time. For all experiments shown, the diverter was deactivated 1 ms before the laser was fired and held off for 15 ms after the laser shot to collect sample-related ions. It was reactivated and prevented background ions from entering for the remaining 75 ms of the ion injection time.

### Evaluation of impact of ESI diverting and NH_4_F doping

**Fig. 1:**
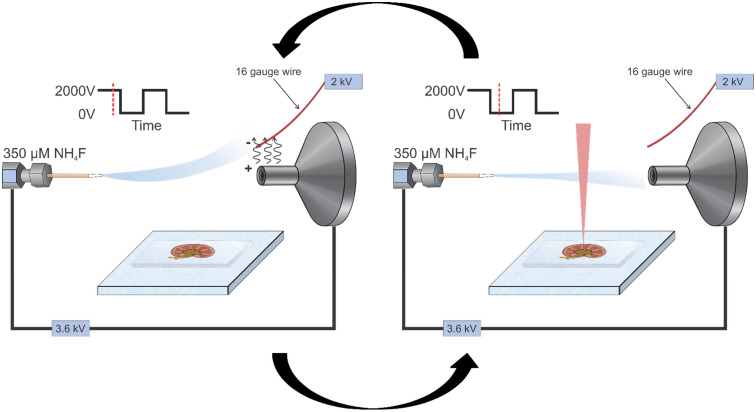
Schematic outlining the process of using ESI-DD for an experiment. For every MS scan performed, the diverter cycles on and off to allow sample related ions to enter and prevent excess background ions based on the laser firing event timing.

Conditions with and without ESI-DD were directly compared for both solutions and tissues (Fig. 1). Control conditions used the control solvent (without NH_4_F) and did not use the ESI diverter, matching typical IR-MALDESI-MSI methods. For ESI-DD, the ESI solvent contained 350 µM NH_4_F and employed the ESI diverter as previously described. To evaluate the impact of ESI-DD on solutions, *N*-linked glycans were cleaved from bovine fetuin as described previously and analyzed in both conditions to compare signal abundance. For MSI, human kidney tissues were prepared, and control and ESI-DD were compared on tissue halves, reducing biological variability by comparing intra-tissue, rather than between multiple tissue slices.

### Data analysis

The raw mass spectra were analyzed in XCalibur (Thermo Fisher Scientific). *N*-Linked glycans were identified by uploading *m*/*z* values to GlycoMod, a theoretical glycan database for generating *N*-linked glycan annotations. GlycoMod can provide a list of *N*-glycans previously reported in literature by linking to GlyConnect. The raw data was also converted to *.mzML through MS Convert (ProteoWizard^32^) and *.imzML^33^ to be analyzed by MSiReader^34,35^ v3.11. All ion images are shown with an associated SMART, a data reporting standard to provide information about the ion image including the step size and laser spot size, molecular identification confidence, annotations, resolving power, and time needed for the acquisition.^36^ All optical images were taken using a Leica LMD 7000 microscope (Leica Microsystems, Wetzlar, Germany).

## Results and discussion

### Analysis of glycans in solution

**Fig. 2:**
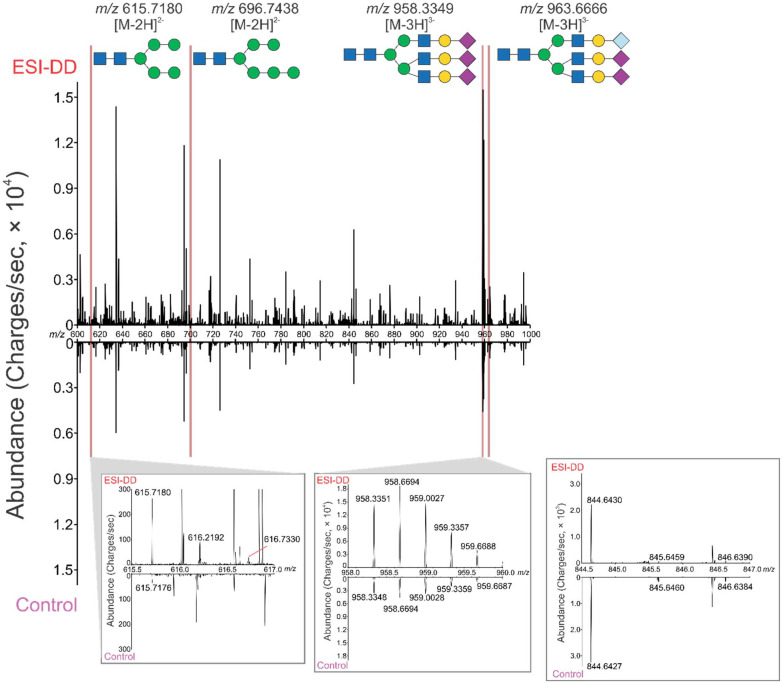
Reflection plot of glycan signals with data from ESI-DD (top) and control (bottom). Four representative glycans and their structures are shown, demonstrating high signal improvement compared to their control counterparts. One low abundant and one high abundant glycan have their isotopic distribution shown, revealing increased abundance in all cases and visualization of previously hidden isotopic peaks. A representative background peak is also shown (*m*/*z* 844.6430), demonstrating decreased abundance in ESI-DD compared to the control.

Initially, the benefits imparted by these techniques were evaluated using a solution of cleaved *N*-linked glycans from bovine fetuin. Wells in a 384 well plate were filled with the prepared solution and analyzed with and without ESI-DD. When using this system, a total of 21 identified glycoforms from the singular glycoprotein were detected (SI Table S1). *N*-Linked glycans were detected with a primary amine terminus due to the use of a basic ammonium bicarbonate buffer, leading to the formation of an amine after cleavage from asparagine residues.^37^Fig. 2 showcases the increased abundance across the *m*/*z* range of sample related ions, while background ions do not show significant increases in signal. Two representative examples of low and high abundant glycans are shown with their isotopic distribution. For low abundant glycans, this raises their signal past the LOD, allowing for their isotopic peaks to be detected while high abundant glycans become more visible and show improved detection frequency. Background ions either show similar or decreased abundance with ESI-DD, shown by a representative background.

**Fig. 3:**
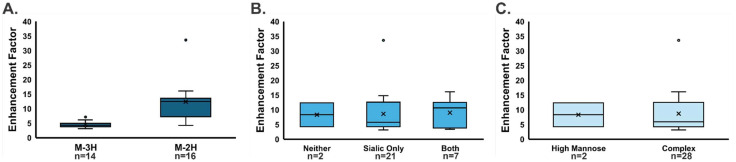
Box and whisker plots show the signal enhancement factor caused by ESI-DD for *N*-glycans cleaved from bovine fetuin, comparing the impact of various factors. An average enhancement factor of 9-fold with a maximum improvement of 33-fold observed. In all cases, no statistically significant effects were detected. (A) Effect of glycan adduct. (B) Effect of the inclusion of either fucose or sialic acid monosaccharides. (C) Effect of glycan type.

To further analyze the spectra and evaluate the average effect on glycan abundance, the ESI-DD abundance was compared against the control abundance to generate an enhancement factor. The enhancements for deprotonated glycans show an average 9-fold enhancement factor and up to 33-fold enhancement was observed (Fig. 3). Moreover, the impact of factors including adduct, the inclusion of fucose or 5-acetylneuraminic acid (sialic acid) monosaccharides, and the type of glycan were investigated (Fig. 3A–C). In all cases, there was no statistically significant difference observed, showing the wide applicability of this technique.

### Analysis of glycans from kidney tissue

**Fig. 4:**
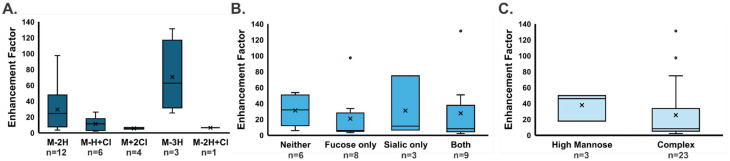
Box and whisker plots evaluating the enhancement factor caused by ESI-DD, comparing the effect caused by various factors. The enhancement ranged from 2-fold to 130-fold, with an average 27-fold enhancement. In all cases, no statistically significant differences were found. (A) Effect of glycan adduct. (B) Effect of the inclusion of fucose or sialic acid monosaccharides. (C) Effect of glycan type.

The system was also evaluated for tissue imaging where kidney slices were divided in half, one half using a control system and the other half with ESI-DD. In the tissue imaging experiment, doubly and triply deprotonated glycans were observed as well as chlorine adducts, likely originating from the citraconic acid buffer (which contains HCl) in the sample preparation process. Among the 15 glycans observed (SI Table S2), many of which were observed in multiple ionization states, enhancement factors ranged from 2-fold to 130-fold improvement in signal, with an average of 27-fold (Fig. 4).

As with bovine fetuin glycans, no significant effects were caused by changes in adduct, the presence of monosaccharides such as fucose or sialic acid, or type of glycan (Fig. 4A–C). Thus, the effect of ESI-DD appears to be relatively agnostic to adduct or small structural changes.

**Fig. 5:**
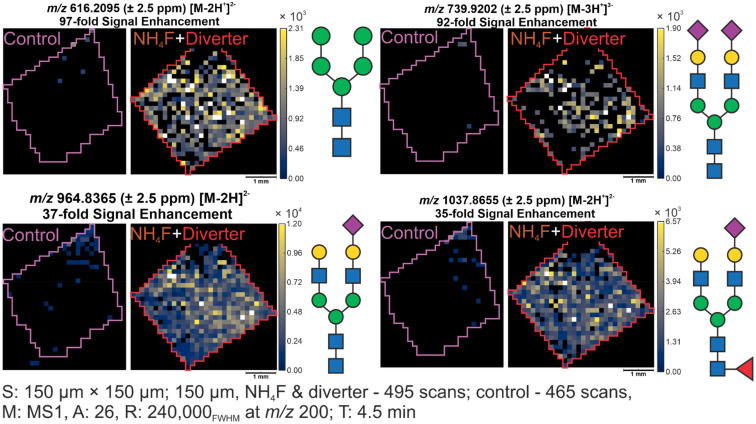
Representative ion heatmaps of glycans enhanced from below the limit of detection to easily observable glycans. ESI-DD and control conditions originate from two halves of the same tissue, confirming the signal enhancement is caused by the techniques used, not by biological variability.

Many glycans observed with ESI-DD were below the limit of detection without these techniques and would not have been detected without their use. Among the 15 glycans, 11 were very significantly enhanced, showing up to two orders of magnitude increase in signal where representative examples are shown in Fig. 5. Heatmaps showing all detected glycans and their signal enhancement are shown in SI Fig. S1. The signal enhancement shown reflects the enhancement in that particular representative tissue sample. Statistical comparisons were made based on the average among the four analyzed tissues. The other four glycans were not as significantly enhanced and were often observed in multiple ionization states. Among the detected glycans, sialic acids were observed without derivatization and glycan structures were confirmed to follow the sialic acid rule.^38,39^

It was surprising that ESI-DD resulted in such a large signal increase across the *m*/*z* range. The initial demonstrations of these techniques individually showed more modest improvements, ranging from 2-fold up to 10-fold improvements. Conversely, many glycans demonstrated two orders of magnitude improvement by the combination of these techniques. The authors propose that this occurred due to their highly orthogonal nature. NH_4_F doping improves signal across the board, including background and sample-related ions. The ESI diverter removes background ions, specifically working to fill the ion trap with analytes of interest. By increasing signal and removing background, we observe a synergistic effect resulting in huge increases in signal, especially for larger analytes.

## Conclusions

The combination of electrospray doping with ammonium fluoride and the application of an electrospray diverter to remove background ions were used for glycan analysis by IR-MALDESI. For solutions of cleaved *N*-glycans, an average signal increase of 9-fold. From tissue, an average of 27-fold increase was seen. In both cases, glycans that were not detected in the control were raised above the limit of detection. This technique is highly valuable for those studying glycomics and utilizing trapping-based instruments to realize high signal gains with minimal setup or instrumentation modification. In the future, this system can be utilized for highly impactful biological studies to map *N*-linked glycans across tissues and between tissue conditions.

## Conflicts of interest

David C. Muddiman is a partial-owner of MSI Software Solutions, LLC. The authors declare no other competing financial interests or any non-financial conflicts.

## Supplementary Material

AN-151-D6AN00090H-s001

## Data Availability

The data presented in this paper can be downloaded from Dryad; the DOI for the dataset is https://doi.org/10.5061/dryad.kkwh70shx. Supplementary information (SI) is available. See DOI: https://doi.org/10.1039/d6an00090h.
