# Supplementary material for: Glycan signal enhancement by ammonium fluoride doping and electrospray diverting
Source: Analyst. 2026 Mar 23;151(9):2704–10. doi: 10.1039/d6an00090h (PMC13037699; doi:10.1039/d6an00090h)

Supplementary Information (SI) for Analyst.
This journal is © The Royal Society of Chemistry 2026

Supporting Information

Glycan Signal Enhancement by Ammonium Fluoride Doping and Electrospray Diverting

Seth M. Eisenberg and *David C. Muddiman

Biological Imaging Laboratory for Disease and Exposure Research (BILDER), Department of Chemistry, North Carolina State University, Raleigh, NC 27695

Submitted: March 9th, 2026

Supplemental Material: 2 Pages / 2 Supplemental Tables

Keywords: IR-MALDESI; Electrospray doping; Electrospray diverting; Glycans; Signal Enhancement;

***Author for Correspondence**

David C. Muddiman, Ph.D.

FTMS Laboratory for Human Health Research

Department of Chemistry

North Carolina State University

Phone: 919-513-0084

Email: dcmuddim@ncsu.edu

Supplemental Table 1. List of glycan masses, compositions, and detected *m/z* originating from prepared bovine fetuin. Glycoform masses refer to the cleaved *N*-linked glycans with an amine terminus and include the addition of water.

Glycoform Mass	Glycan Composition	$[M-2H]^{2-}$	$[M-3H]^{3-}$
1233.4494	$Hex_5HexNAc_2$	615.7174	-
1395.5022	$Hex_6HexNAc_2$	696.7438	-
1727.6242	$Hex_5HexNAc_3NeuAc_1$	862.8048	-
1889.6770	$Hex_6HexNAc_3NeuAc_1$	943.8312	-
1930.7036	$Hex_5HexNAc_4NeuAc_1$	964.3445	642.5606
2221.7990	$Hex_5HexNAc_4NeuAc_2$	1109.8922	739.5924
2237.7939	$Hex_5HexNAc_4NeuAc_1NeuGc_1$	1117.8897	-
2238.8143	$Hex_6HexNAc_4Fuc_1NeuAc_1$	1118.3999	-
2367.8569	$Hex_5HexNAc_4Fuc_1NeuAc_2$	1182.9212	-
2512.8944	$Hex_5HexNAc_4NeuAc_3$	1255.4399	836.6242

2586.9312	Hex$_6$HexNAc$_5$NeuAc$_2$	1292.4583	861.3031
2732.9891	Hex$_6$HexNAc$_5$Fuc$_1$NeuAc$_2$	1365.4873	909.9891
2878.0266	Hex$_6$HexNAc$_5$NeuAc$_3$	1438.0060	958.3349
2894.0215	Hex$_6$HexNAc$_5$NeuAc$_2$NeuGc$_1$	1446.0035	963.6666
3169.1220	Hex$_6$HexNAc$_5$NeuAc$_4$	1583.5537	1055.3667
2092.7564	Hex$_6$HexNAc$_4$NeuAc$_1$	-	696.5782
2894.0467	Hex$_5$HexNAc$_3$Fuc$_4$NeuAc$_3$	-	963.6749
3024.0845	Hex$_6$HexNAc$_5$Fuc$_1$NeuAc$_3$	-	1007.0209
3185.1169	Hex$_6$HexNAc$_5$NeuAc$_3$NeuGc$_1$	-	1060.6984
3460.2174	Hex$_6$HexNAc$_5$NeuAc$_5$	-	1152.3985
3534.2542	Hex$_7$HexNAc$_6$NeuAc$_4$	-	1177.0775

Supplemental Table 2. List of glycan masses, compositions, and detected *m/z* originating from prepared FFPE human kidney tissues. Glycoform masses refer to the cleaved *N*-linked glycans with an alcohol terminus and include the addition of water.

Glycoform Mass	Glycan Composition	$[M-2H]^{2-}$	$[M-H+Cl]^{2-}$	$[M+2Cl]^{2-}$	$[M-3H]^{3-}$	$[M-2H+Cl]^{3-}$
1234.4336	Hex$_5$HexNAc$_2$	616.2095	634.1978			
1396.4866	Hex$_6$HexNAc$_2$	697.2360	837.2773			
1640.5926	Hex$_5$HexNAc$_4$	819.2890		855.2657		
1786.6506	Hex$_5$HexNAc$_4$ Fuc$_1$	892.3180	910.3063	928.2947		
1827.6766	Hex$_4$HexNAc$_5$ Fuc$_1$	912.8310		948.8083		
1931.6876	Hex$_4$HexNAc$_5$ Fuc$_1$NeuGc$_1$	964.8365	982.8248			
1989.7296	Hex$_5$HexNAc$_5$ Fuc$_1$	993.8575		1029.8348		
2077.7456	Hex$_5$HexNAc$_4$ Fuc$_1$NeuAc$_1$	1037.8655	1055.8538		691.5746	
2222.7826	Hex$_5$HexNAc$_5$ NeuAc$_2$	1110.3840			739.9202	
2262.8589	Hex$_6$HexNAc$_5$ NeuAc$_1$		1165.3916			
2368.8406	Hex$_5$HexNAc$_4$ Fuc$_1$NeuAc$_2$	1183.4130			788.6062	751.9124
2442.8776	Hex$_6$HexNAc$_5$ Fuc$_1$NeuAc$_1$	1220.4315	1238.4208			
2735.9899	Hex$_6$HexNAc$_5$ Fuc$_1$NeuAc$_2$	1365.9804				
2589.9305	Hex$_6$HexNAc$_5$ NeuAc$_2$	1292.9507				
3031.1149	Hex$_6$HexNAc$_5$ Fuc$_1$NeuAc$_3$				1007.3498	

Supplemental Figure 1. Heatmaps of all glycans observed in kidney tissue, comparing two halves of the same tissue. One half was imaged using control conditions while the other used ESI-DD, observing a range of signal enhancements. The signal enhancement reported here refers to the enhancement of the representative tissue shown. Proposed glycan structures are provided by GlyConnect based on reported MS/MS of the experimentally observed *m/z* peaks.

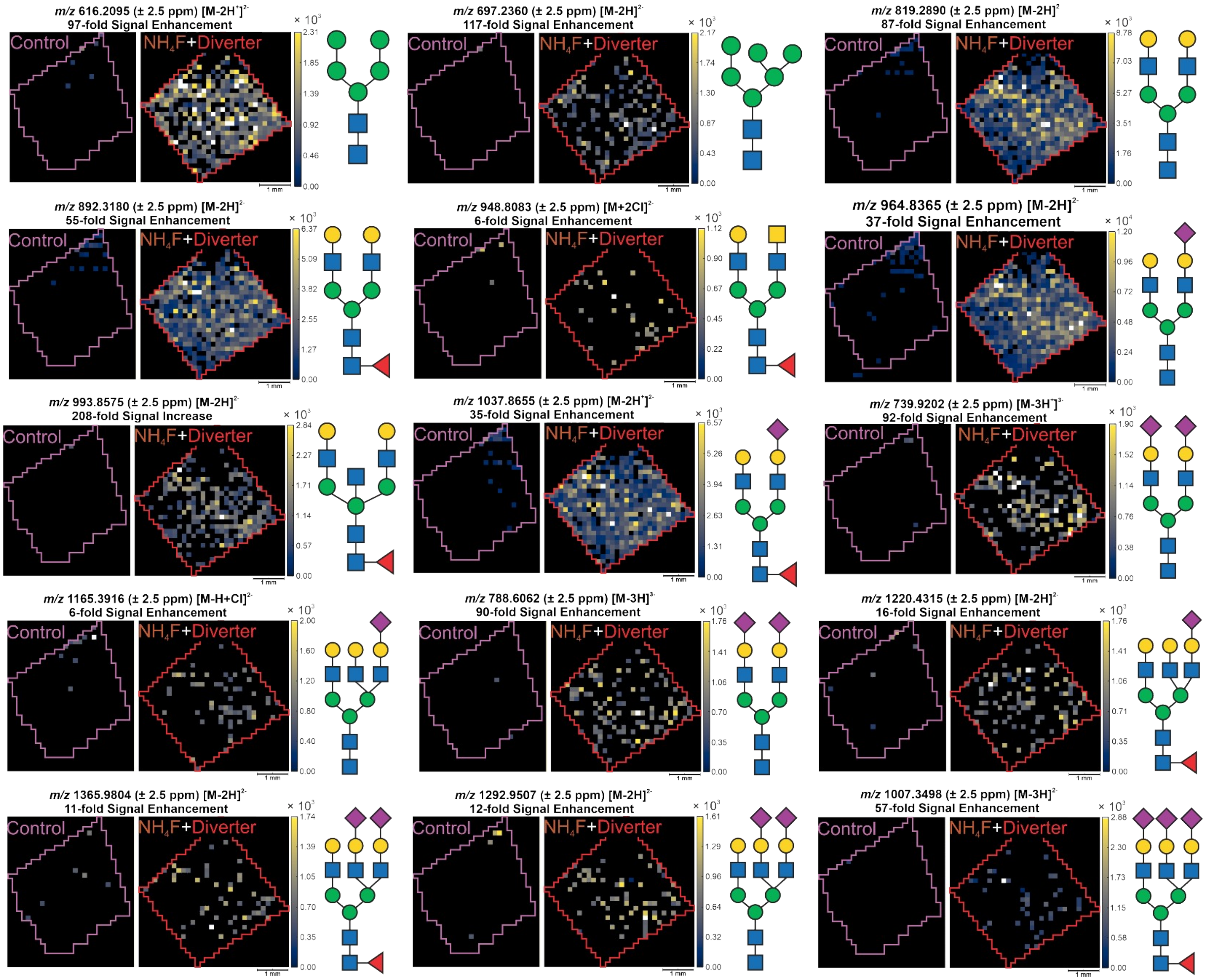

Supplement: AN-151-D6AN00090H-s001 [file AN-151-D6AN00090H-s001.pdf]
